# The Effect of Plant Additives on the Stability of Polyphenols in Dried Black Chokeberry (*Aronia melanocarpa*) Fruit

**DOI:** 10.3390/foods10010044

**Published:** 2020-12-26

**Authors:** Andrzej Sidor, Agnieszka Drożdżyńska, Anna Brzozowska, Anna Gramza-Michałowska

**Affiliations:** 1 Department of Gastronomy Sciences and Functional Foods, Faculty of Food Science and Nutrition, Poznań University of Life Sciences, Wojska Polskiego 31, 60624 Poznań, Poland; andrzej.sidor@up.poznan.pl (A.S.); anna.brzozowska@up.poznan.pl (A.B.); 2 Department of Biotechnology and Food Microbiology, Faculty of Food Science and Nutrition, Poznań University of Life Sciences, Wojska Polskiego 31, 60624 Poznań, Poland; agnieszka.drozdzynska@up.poznan.pl

**Keywords:** black chokeberry, *Aronia melanocarpa*, cinnamon, *Cinnamomum*, clove, *Syzygium aromaticum*, fruits, drying method, polyphenols, bioactive compounds

## Abstract

Chokeberry fruit exhibits a high level of pro-health potential, associated with a significant amount of polyphenol content and antioxidant activity. The fruit is easily perishable and therefore needs to be processed in short order to ensure its availability throughout the year. For this purpose, the fruit is dried, which has an impact on the preservation of bioactive components such as polyphenols. In the study, chokeberry fruit was influenced by a suspension of spices, including clove and cinnamon, and subsequently dried using the freeze-drying, convection, and microwave radiation methods. Freeze-drying was concluded to be the most desirable method of drying, which preserves the largest amount of polyphenols while soaking fruit in a suspension of cinnamon or cloves accelerated the loss of anthocyanins during the storage period. The obtained test results may constitute a valuable source of information for manufacturers in designing new products with increased pro-health potential, whose properties result from the high content of polyphenols and other plant additives.

## 1. Introduction

Chokeberry fruit exhibits a high pro-health potential, which may help in the prevention, as well as during the course of civilization diseases, such as diabetes, hypertension, inflammation, cancer, and others [1,2,3]. The pro-health activity of chokeberry fruit is often associated with a high content of polyphenols (anthocyanins, proanthocyanidins, phenolic acids, flavonols) and antioxidant properties [4,5,6,7,8,9]. Chokeberry fruit production is seasonal, ensuring its availability throughout the year requires processing [10]. One of the methods for extending the durability of fruit is drying. The method of fruit drying, as well as the process of preparation for drying, affects the preservation of bioactive components such as polyphenols. Most frequently used drying methods consist of convection and freeze-drying, while the new direction of research involves the use of microwave and infrared radiations, radio frequency drying, methods using reduced atmospheric pressure, or differences in osmotic pressure, as well as combined methods. Convection drying is a process of removing water from the material by exposing it to a hot air stream. Increased temperature of the material leads to water evaporation, as well as contributing to significant changes in the chemical and physical properties of food. Most frequently, the level of undesirable changes increases with the raise of the process temperature. Freeze-drying consists of the sublimation of water crystals from the frozen material under reduced pressure, influenced by the supplied heat energy. The material is kept in a frozen state, which allows water to evaporate in layers by moving the ice boundary towards the center of the material. Freeze-drying, to a large extent, prevents damage to the structure of the dried material and allows for considerable preservation of the components in an unaltered state. The combined method, involving the pre-drying of the material and its subsequent drying, using the microwave radiation energy in a vacuum, allows for relatively good preservation of bioactive components. Convection pre-drying enables the removal of the unbound part of water. Microwave drying in a vacuum allows significantly increasing the efficiency and shortening the processing time [11,12].

It may be beneficial to prepare the raw material for drying, by applying spices with strong antioxidant properties, in order to protect the products from polyphenol losses. Cinnamon or cloves may act as an additional source of polyphenols. However, there is no clear evidence to confirm or deny that the addition of antioxidants from other sources contributes to the protection of compounds natively found in the dried material. Therefore, research on the unpredictable effect of the interaction between the polyphenols from different sources should not be overlooked. Moreover, the addition of highly antioxidant raw materials, such as cinnamon and cloves, to the fruit prior to drying may increase its pro-health potential. Cinnamon exhibits an anti-diabetic, anti-inflammatory, anti-obesity, anti-tumor, antibacterial, antiviral, cardiovascular protective, cytoprotective, neuroprotective, and immunoregulatory activity [13]. The pharmaceutical purposes of cloves include the antimicrobial (antibacterial, antifungal, anticandidal), antiviral, anti-inflammatory, and anti-tumor effects [14,15].

The unpredictable effect of increasing or inhibiting the activity of a different active compound mixture is often overlooked in combinations of raw materials rich in bioactive ingredients, including polyphenols from different sources. To the best of our knowledge, there is no research on changes in the polyphenol content during the storage of berries. The conducted study provides information regarding the possibility of selecting a drying method based on the expected level of preservation of bioactive compounds in the form of polyphenols, after drying and during the storage period. It further contributes to the knowledge of the mutual influence of raw materials used, i.e., chokeberry combined with cinnamon or cloves. The obtained test results may constitute a valuable source of information for manufacturers in designing new products with increased pro-health potential, which properties result from the high content of polyphenols.

## 2. Materials and Methods

### 2.1. Reagents

Chemical reagents (acetonitrile, L-ascorbic acid, methanol, acetic acid, and formic acid) were purchased from Sigma-Merck, Poznań, Poland. For the qualitative and quantitative content of polyphenols the following standards were used: gallic acid, caffeic acid, isovanilic acid, chlorogenic acid, neochlorogenic acid, p-coumaric acid, 4-hydroxybenzoic acid, 3,4-dihydroxybenzoic acid, orientin (luteolin-8-glucoside), quercetin, quercetin-3-O-glucoside, procyanidin B2, luteolin, kaempferol (Sigma-Aldrich, Poznań, Poland), cyanidin-3-O-glucoside, cyanidin-3-O-galactoside, cyanidin-3-O-arabinoside (Polyphenols Laboratories AS, Sandnes, Norway), cyanidin-3-O-xyloside (Toronto Research Chemicals, North York, ON, Canada), quercetin-3-O-galactoside, quercetin-3-O-vicianoside, quercetin-3-O-rutinoside, (+)-catechin and (−)-epicatechin (Extrasynthese, Genay, France). All other chemicals were of analytical grade.

### 2.2. Plant Material

The material for the study consisted of black chokeberry fruit (Aronia melanocarpa), purchased from the Agricultural and Fruit-growing Experimental Farm (Przybroda, Poland), as well as the spice plants available in the retail trade in the form of Kotányi cinnamon (KOTÁNYI Polonia, Warszawa, Poland) and Kamis cloves (McCormick Polska, Stefanowo, Poland). The fruit was evaluated according to the guidelines of the Polish PN-R-75032:1996 standard [16] and qualified as a class extra.

### 2.3. Sample Preparation

After harvesting, the fruit was subjected to the process of cleaning, separation, and washing. Prior to drying, it was soaked for 12 h in distilled water and 5% aqueous suspension of cinnamon or cloves (1:1). The concentration of the aqueous spice suspensions was determined on the basis of sensory analysis. The highest concentration value was adopted, at which no deterioration of the sensory quality of the final product was observed. Taste was the main and required differentiating factor to be taken into account. The prepared fruit was dried, using the freeze-drying, convection, and microwaving methods. The fruit freeze-drying was conducted with the use of the Alpha 1-4 LSC (Martin Christ GmbH, Osterode am Harz, Germany) freeze-dryer, at 0.960 kPa, the condenser temperature of (−60 °C), as well as the shelf temperature of 25 °C [17]. Convection drying was carried out in the STREA 7 fluid bed dryer (Aeromatic AG, Bubendorf, Switzerland). The process parameters were selected by carrying out subsequent trials at different temperatures. Fruits with the best sensory evaluation were selected for further steps of analysis. The air temperature at the entrance point reached 78–80 °C, while at the exit 64–68 °C. The weight of the charge amounted to 10 kg of fruit at a time. Fruit was dried to 24 ± 1% of water content. Microwave drying in conditions of reduced pressure was conducted with a laboratory kit for drying biological materials using microwaves in a vacuum. Microwave drying under reduced pressure was carried out on fruit conventionally pre-dried to 24 ± 1% of water content. The conditions of microwave drying under reduced pressure were selected as a result of subsequent tests, involving the modification of the drying parameters, number of stages, as well as power and microwave radiation time. The fruits with top-rated sensory evaluation were selected for further steps of research. The process of proper drying was conducted in three stages: stage 1: the microwave power of 1500 W in 40 s; stage 2: 500 W, 60 s; stage 3: 1500 W, 10 s. The final water activity of dried fruits was 0.421–0.514 (unpublished data). Dried fruit, which had not been subjected to the previous soaking process, was a control sample, hereinafter referred to as no preparation. Dried fruits were divided into 100 g packages, placed in paper bags, and stored for nine months (analysis frequency every 3 months) without light in the air-conditioned room at a temperature of 20 °C. Significant fluctuations in antioxidant activity occur during the storage of various products, including fruit. The results presented in this paper are part of an experiment aimed at the determination of the antioxidant activity of dried fruit, therefore it was decided to check to what extent it is related to changes in the content of polyphenols (unpublished data, subject of another paper).

### 2.4. HPLC Analysis of Chokeberry Polyphenolic Compounds

The polyphenolic compounds for chromatographic analysis were extracted with an extraction mixture, which consisted of 150 mL HPLC grade methanol ≥99.9%, 345 mL deionized water, 0.5 mL HPLC grade acetic acid ≥99.7%, and 1 g ascorbic acid [18]. Seven milliliters of the extraction mixture was added to 0.5 g of crushed fruit and sonicated in a Sonic 2 ultrasonic cleaner (POLSONIC, Warszawa, Poland) for 15 min. The mixture was stored for 24 h at 4 °C and it was sonicated for 15 min again [4]. The extracts were purified by passing through 0.45 µm PTFE syringe filters.

The identification of chokeberry fruit polyphenols was performed on a chromatography system (Agilent Technologies, Santa Clara, CA, USA) equipped with a pump (G1312B), diode detector (G1315C), and autosampler (G1329B) [10]. The separation was performed on an SB-C18 column (50 mm × 4.6 mm, 1.8 µm particle diameter, Agilent Technologies, Santa Clara, CA, USA). The mobile phase was comprised of (A) 4.5% formic acid (LC-MS grade 99%) and (B) acetonitrile (HPLC grade ≥99.9%). A 10 µL fruit extract sample was directly injected into the system. The gradient program was as follows: 0 min 3% B, 7 min 9% B, 13 min 12% B, 20 min 14% B, 21 min 80% B, 26 min 80% B, 27 min 3% B, 36min 3% B. The flow rate was 1 mL/min and the column was maintained at 25 °C. Detection of flavanols was accomplished at 280 nm, phenolic acids at 320 nm, flavonols at 360 nm, and anthocyanins at 520 nm. The elaboration of the chromatographic data was carried out on a ChemStation for LC 3D system software (Agilent Technologies, Santa Clara, CA, USA). Standards were used to compare spectra and retention times for compound identification and quantitative calculations. Results are expressed in mg/100 g FW (fresh weight) for fresh fruits and in mg/100 g of dried fruits for the fruits subjected to a drying process.

### 2.5. Analysis of Polyphenolic Compounds of Spice Plants in the LC-MS System

Spice phenolic compounds analyses were performed on Dionex UltiMate 3000 UHPLC system (Thermo Fisher Scientific, Waltham, MA, USA) equipped with reversed-phase Kinetex™ 1.7 µm C18 100 Å, 100 × 2.1 mm column (Phenomenex, Torrance, CA, USA) [10]. The method used a gradient starting with a mixture of 95% solvent A (0.1% formic acid in water) to 5% solvent B (acetonitrile ≥99.9%) and increased linearly over the course of 20 min to 5% solvent A and 95% solvent B. An injection volume of 3 µL was used, the flow rate was set at 0.2 mL/min and the column was kept at a temperature of 40 °C. The chromatography system was coupled with a Bruker maXis ultra-high-resolution tandem mass spectrometer (Bruker Daltonik, Bremen, Germany) using a quadrupole and a time-of-flight analyzer. For identification polyphenols ESI-MS was used employing the following parameters: capillary voltage 4500 V, nitrogen nebulization at 1.8 bar pressure, drying gas flow (N_2_) 9 L/min at 200 °C, scan range 80–1200 *m*/*z*. The ESI-MS system was calibrated with sodium formate salt, the molecular weight standard was dosed each time at the beginning of the chromatographic separation. The results were developed using Data Analysis 4.1 (Bruker Daltonik, Bremen, Germany). The compounds identification was carried out by comparing their retention times and mass spectra with standards. Quantitative analyses were carried out based on prepared standard curves and the results were presented as mg/100 g of spice.

### 2.6. Anthocyanin Degradation Kinetics

Cyanidin-3-O-arabinoside, cyanidin-3-O-xyloside, cyanidin-3-O-galactoside, and cyanidin-3-O-glucoside degradation over time for each treatment, and the cyanidins half-life in each mixture was calculated using the previously described equation [10].

### 2.7. Statistical Analysis

Data were expressed as the mean ± standard deviation. A one-way analysis of variance (ANOVA) followed by Tukey HSD post hoc test was used to analyze the significant differences between the groups. Effect of cinnamon or cloves on polyphenols stability after addition to fruits and drying process, next during storage was studied. A value of *p* < 0.05 was considered statistically significant. Software used for the ANOVA test was Statistica 13 (StatSoft Polska, Kraków, Poland). Reaction rates for cyanidin glycosides degradation were obtained from linear regression conducted using Microsoft Excel 2016 (Redmond, WA, USA).

## 3. Results and Discussion

The study examined the influence of the drying method (freeze-drying, combined, and convection), as well as the impact of storage time on variations in the content of polyphenolic compounds in chokeberry dried fruit. The raw material intended for drying consisted of fresh chokeberry fruit without additions, as well as fruit with cinnamon or cloves. Fruit with additives was prepared for drying by soaking in aqueous suspensions of cinnamon or cloves. The obtained dried fruit was stored and periodically analyzed in order to monitor changes in the content of selected anthocyanins, phenolic acids, flavonols, and flavanols.

### 3.1. Profiles of Polyphenolic Compounds in Raw Chokeberry Fruit

The analysis of the polyphenolic composition of fresh chokeberry fruit allowed us to determine elements such as phenolic acids, flavanols, flavonols, and anthocyanins. The main group of established polyphenolic compounds consisted of cyanidin-3-O-galactoside, arabinoside, glucoside, and xyloside anthocyanins, which the content amounted to 600.13; 406.75; 27.01, and 56.25 mg/100 g of fresh weight (FW) (Table 1). The phenolic acid content was relatively high (Table 2). Neochlorogenic acid occurred in fresh fruit (67.96 mg/100 g) in a smaller amount than chlorogenic acid (96.35 mg/100 g FW). Fresh fruit contained flavanols (catechin and epicatechin) amounting to 20.42 mg/100 g FW (Table 3). A small part of polyphenols comprised of flavonols, which were quercetin glycosides (Table 4).

The approximate content of anthocyanins in fresh fruit was determined by Wu et al. [8] cyanidin-3-O-arabinoside, glucoside, and xyloside amounted to 399.3, 37.6, and 51.5 mg/100 g FW, while cyanidin-3-O-galactoside occurred in a much higher quantity of 989.7 mg/100 g FW.

Some of the black chokeberry fruit analyzed by Ochmian, Grajkowski, and Smolik [19] had a particularly similar composition of chlorogenic acid—96.6 mg/100 g FW; neochlorogenic acid—59.3 mg/100 g FW, as well as quercetin-3-O-galactoside, glucoside, rutinoside, and vicianoside—9.9, 7.1, 5.50 and 3.3 mg/100 g FW respectively. The content of cyanidin-3-O-galactoside (636.0 mg/100 g FW) and cyanidin-3-O-glucoside (27.2 mg/100 g FW) oscillated around a comparable level, whereas cyanidin-3-O-arabinoside (299.4 mg/100 g FW) and cyanidin-3-O-xyloside (38.2 mg/100 g FW) occurred at a lower level.

Similarities were also found in the content of the polyphenolic compounds, indicated in chokeberry fruit derived from different crops. When determining the ranges (minimum-maximum), a similar or comparable level of content was recorded for chlorogenic acid—83.97–110.62 mg/100 g FW, neochlorogenic acid—74.60–99.76 mg/100 g FW, epicatechin—15.76–32.18 mg/100 g FW, quercetin-3-O-galactoside, glucoside, rutinoside, and vicianoside—9.91–14.57, 7.07–8.87, 5.50–6.27 and 3.84–5.41 mg/100 g FW respectively. Cyanidin was present to a smaller extent, while its maximum content amounted to: cyanidin-3-O-galactoside—515.22 mg/100 g FW, cyanidin-3-O-arabinoside—249.46 mg/100 g FW, cyanidin-3-O-glucoside—21.51 mg/100 g FW, and cyanidin-3-O-xyloside—33.39 mg/100 g FW [20].

### 3.2. Profiles of Polyphenolic Compounds in Cinnamon and Cloves

Determination of the polyphenol content in cinnamon indicated the presence of: 3,4-dihydroxybenzoic acid (5.79 mg/100 g), chlorogenic acid (4.63 mg/100 g), catechin (0.92 mg/100 g) and procyanidin B2 (1.84 mg/100 g). The cloves revealed more diverse profiles of polyphenols: caffeic acid (3.46 mg/100 g), chlorogenic acid (169.51 mg/100 g), gallic acid (79.5 mg/100 g), isovanillic acid (1.13 mg/100 g), p-coumaric acid (1.30 mg/100 g), kaempferol (5.24 mg/100 g), luteolin (2.12 mg/100 g), orientin (1.69 mg/100 g), quercetin (5.54 mg/100 g), quercetin-3-D-galactoside (2.42 mg/100 g) and naringenin (0.56 mg/100 g).

By studying the composition of cinnamon, Al-Numair, Ahmad, Ahmed, and Al-Assaf [21] were able to determine a greater number of flavonoids, including rutin (0.672 mg/100 g), quercetin (0.172 mg/100 g), kaempferol (0.016 mg/100 g), isorhamnetin (0.103 mg/100 g), and catechin (1.9 mg/100 g). Luo et al. [22] found the non-flavonoid phenolic compounds in cinnamon. A more numerous group of phenolic acids occurring in cinnamon was indicated by Klejdus and Kováčik [23].

Various polyphenolic compounds, such as gallic, caffeic, and syringic acid [24], tamarixetin-3-O-β-d-glucopyranoside, ombuin-3-O-β-d-glucopyranoside, and quercetin [25], as well as ellagitannins, such as casuarictin, eugenin, tellimagrandin I, and 1,3-di-O-galloyl-4,6-O-[S]-hexahydroxydiphenoyl-β-d-glucose have been determined in cloves [26]. Following the extraction of clove oil, Gupta and Prakash [27] identified biflorin, kaempferol, rhamnocitrin, myricetin, gallic acid, and ellagic acid in the material.

### 3.3. Content of the Polyphenolic Compounds in Chokeberry Fruit after the Process of Drying

Chokeberry fruit was dried, using the commonly applied convection and freeze-drying methods, as well as the combined, convection-microwave-vacuum method, which belongs to the group of innovative, increasingly often used in practice methods. The applied methods differ with regard to the cost of the drying process and the quality of the resulting dried fruit, e.g., the preservation of bioactive components and structure. The low temperature and gradual evaporation of subsequent layers of water from the frozen material during the process of freeze-drying allow for obtaining a well-preserved structure and relatively insignificant losses of polyphenolic compounds. Subjecting the raw material to the stream of heated air in convection drying leads to a significant shrinkage of the dried material and a large loss of thermolabile components. Convection-microwave-vacuum drying provides the possibility to maintain a better structure and reduce the interaction time between the increased temperature and the raw material, compared to convection drying [28,29].

Predominant anthocyanins of dried fruit comprised of cyanidin-3-O-galactoside and cyanidin-3-O-arabinoside, whereas cyanidin-3-O-xyloside and cyanidin-3-O-glucoside occurred in smaller amounts (Table 1). The method of drying significantly influenced the anthocyanin content. Taking into account the preservation of the highest possible amount of anthocyanins, freeze-drying should be used. The content of cyanidin-3-O-galactoside in freeze-dried products following the process of drying amounted to 1403–1611 mg/100 g; cyanidin-3-O-arabinoside to 996–1112 mg/100 g; cyanidin-3-O-xyloside to 120.8–139.6 mg/100 g, and cyanidin-3-O-glucoside to 51.14–60.68 mg/100 g. Fruit dried using the convection and combined methods had about three times less cyanidin-3-O-galactoside, and over four times less cyanidin-3-O-arabinoside, xyloside, and glucoside content in relation to freeze-dried fruit. The addition of cinnamon or cloves prior to fruit drying resulted in higher losses of cyanidin during the combined and convection drying.

Freeze-dried fruit contained between 199.6–208.9 mg/100 g of neochlorogenic acid and 271.1–297.3 of chlorogenic acid (Table 2). Combined method of drying allowed to obtain raw material with neochlorogenic and chlorogenic acid content of 160.0–179.2 and 236.7–273.2 mg/100 g respectively. The lowest concentration of both acids was determined in convection-dried fruit (142.4 and 200.2 mg/100 g).

Dried fruit contained catechin and epicatechin, while the concentration of the compounds varied, depending on the drying method (Table 3). The highest level of epicatechin was recorded in freeze-dried fruit (60.26–63.26 mg/100 g), followed by fruit dried using the combined (40.8–46.29 mg/100 g) and convection methods (30.77–37.56 mg/100 g). Catechin was present in smaller amounts, while its content in all fruit, regardless of the drying method and additives, fell within the range of 11.95–23.01 mg/100 g. The statistical analysis allowed us to conclude that the content of catechin in fruit subjected to different preparation prior to drying did not differ significantly within a given drying method. 

An analogous situation occurred in the case of flavanols in dried chokeberry fruit (Table 4). Among flavonols, quercetin-3-O-galactoside was present in the largest amount, quercetin-3-O-rutinoside and quercetin-3-O-glucoside occurred at a similar level, while quercetin-3-O-vicianoside appeared in the smallest amount. Freeze-dried, as well as dried using the combined method fruit contained similar content of flavonols quercetin-3-O-galactoside (29.28–36.93 mg/100 g), rutinoside (22.13–26.98 mg/100 g), glucoside (21.37–27.20 mg/100 g), and vicianoside (18.45–21.10 mg/100 g). A smaller amount of flavonols was determined in convection dried fruits—quercetin-3-O-galactoside (20.58–26.16 mg/100 g), rutinoside (16.48–19.57 mg/100 g), glucoside (16.22–19.74 mg/100 g), and vicianoside (13.57–15.62 mg/100 g).

The influence of the drying method on the preservation of polyphenols was defined regardless of the sample preparation method. The most beneficial drying method for the preservation of polyphenols was freeze-drying (3384 mg/100 g), while the content of polyphenols in fruits dried by combined and convection methods was significantly lower (1130 and 1101 mg/100 g). This relation also applied to anthocyanins, which occurred at the level of 2717, 548, and 611 mg/100 g in fruit dried by freeze-drying, combined, and convection methods.

There were significant differences between freeze-dried, dried by the combined and convection methods fruit with regard to phenolic acid (486.3, 421.7, and 364.0 mg/100 g) and flavanol content (77.18, 65.10, and 50.45 mg/100 g). Freeze-dried and dried using the combined method fruit contained a statistically consistent amount of total flavonols, while their content in convection dried fruit was significantly lower—104.1, 95.48, and 76.15 mg/100 g respectively.

The method of drying has a significant impact on the polyphenol content of dried fruit. Samoticha, Wojdyło and Lech [17] obtained the highest total content of anthocyanins and polyphenols in freeze-dried fruit’s dry weight (2227 and 7265 mg/100 g DW). Convection drying (781–965 and 4956–5631 mg/100 g DW) and microwave-vacuum drying (1797–2076 and 4954–5597 mg/100 g DW) proved to be the least beneficial, while a relatively good effect was obtained by convection-vacuum-microwave drying (1458–2208 and 5697–6554 mg/100 g DW). The drying method and parameters influenced the preservation of polyphenols in jujube fruit. The largest amount of polyphenols was preserved by freeze-dried fruit, followed by microwave-vacuum, convection-vacuum-microwave, and convection method dried fruit [29].

Lower losses of anthocyanin in raspberry, boysenberry, redcurrant, and blackcurrant occurred during the freeze-drying process than during the convection drying [30]. Freeze-drying proved to be a more beneficial method of chokeberry drying. The level of preservation of the total number of polyphenols, flavonoids, and anthocyanins, was the highest in the freeze-drying process (919.7, 66.1, 3715 mg/g). Convection drying allowed for greater preservation of flavonoids and anthocyanins (58.5, 2231 mg/g) than sun drying (52.0, 1146 mg/g). However, sun drying the chokeberry fruit allowed for preserving a higher total number of polyphenols (876.4 mg/g) than convection drying (792.3 mg/g) [31].

Sadowska et al. [32] produced chokeberry powders using various drying methods. Taking into account the content of the total number of polyphenols, fluid bed drying proved to be the most effective method to obtain chokeberry powders (2484 mg GAE/100 g DW), the powders achieved using freeze-drying, vacuum drying at 65 °C and convection drying at 70 °C statistically did not differ in a significant way and contained 2256, 2149 and 2147 mg GAE/100 g DW respectively. The total amount of cyanidin-3,5-digalactoside and cyanidin-3,5-diarabinoside content was the highest in fluid bed dried powder (1087 mg/100 g DW), and the lowest in convection dried powder (422 mg/100 g DW), while freeze-dried and vacuum dried powders contained 904.23 and 845.58 mg of the determined anthocyanins per 100 g of dry mass. A relevant factor influencing the preservation of polyphenols in dried chokeberry fruit is the drying temperature. The increase of the convection drying temperature from 50 °C to 60 °C and 70 °C, resulted in a decrease in the total content of anthocyanins, flavonoids, and polyphenols [33].

### 3.4. The Influence of Plant Extracts on the Content Anthocyanins in Stored Dried Chokeberry Fruit

Dried fruit packed in paper bags was stored at a temp. of 20 °C. Samples for analysis were taken from 3 measurement points—after drying, as well as after 3 and 9 months of storage. Anthocyanins may be ranked among the most labile polyphenols in dried chokeberry fruit. During storage, fruit with cinnamon or cloves decomposed the most rapidly (Table 5). The constant speed of anthocyanin degradation, depending on cyanidine glycosides and drying method amounted to k = 0.6–1.7 × 10^−3^ [d^−1^]. It should be noted, that the constant rates of cyanidin degradation in freeze-dried fruit (k = 0.6–0.9 × 10^−3^ [d^−1^].) were lower than in fruit dried using the convection and the combined methods, particularly for freeze-dried fruit without additives, e.g., for cyanidin-3-O-galactoside, k = 0.6 × 10^−3^ [d^−1^]. Spices added to fruit prior to freeze-drying were a factor that accelerated the loss of these compounds. The chokeberry anthocyanins half-life period of t1/2, determined on the basis of modifications in the concentration level during fruit storage, was relatively long. The shortest period t1/2 amounted to no less than 400 days. The longest period t1/2 was indicated for cyanidin-3-O-galactoside in washed, freeze-dried fruit (1155 days). Cyanidin-3-O-galactoside proved to be one of the most durable anthocyanins. Changes in freeze-dried fruit, caused by the influence of cinnamon and cloves (cyanidin-3-O-galactoside—t1/2 = 866 and 770, cyanidin-3-O-arabinoside—t1/2 = 630 and 630, cyanidin-3-O-xyloside—t1/2 = 495 and 578 and cyanidin-3-O-glucoside—t1/2 = 533 and 495 days), occurred slower than in fruit dried using the combined and convection methods.

Quantitative losses of anthocyanins during storage were the highest in freeze-dried fruit for cyanidin-3-O-galactoside and cyanidin-3-O-arabinoside (Table 1). The concentration of cyanidin-3-O-galactoside was reduced from 434.3 mg/100 g (washed fruit) to 685.4 mg/100 g (fruit with the addition of cloves). Cyanidin-3-O-arabinoside was degraded from 401.9 mg/100 g (washed fruit) to 571.5 mg/100 g (fruit with the addition of cloves). The losses of individual cyanidin-3-O-xyloside, as well as glucoside and anthocyanins in stored fruit after convection and the combined method drying, were reduced due to the lower content of the compounds in the initial stage of the experiment, e.g., 5.16–6.71 mg/100 g for cyanidin-3-O-glucoside in convection dried fruit.

### 3.5. The Influence of Plant Extracts on the Content Phenolic Acids in Stored Dried Chokeberry Fruit

The concentration of neochlorogenic acid in the fruit was being noticeably reduced under the influence of cloves in freeze-dried, as well as dried by the combined method fruit throughout the entire storage period (Table 2). After three months, the changes in the neochlorogenic acid content of most fruit were not significant. The next stage of storage resulted in a decrease in neochlorogenic acid concentration in all cases (8.70–38.04 mg/100 g). Losses of neochlorogenic acid throughout the entire storage period involved fruit with cinnamon and cloves, amounting to 15.7 and 18.0%, 22.1 and 23.0%, 5.8 and 16.2% for freeze-drying, combined method drying, and convection drying respectively.

The content of chlorogenic acid in freeze-dried chokeberry fruit decreased after three and six months of storage. Regardless of the additives involved, all freeze-dried fruit contained a similar, statistically comparable amount of chlorogenic acid (232.9–249.8 mg/100 g). After three months of storage of convection dried chokeberry fruit, a statistically insignificant increase of chlorogenic acid concentration was recorded, while after nine months of storage, a decrease of its concentration followed. At the end of the study, chokeberry fruit with the addition of cloves contained the lowest level of chlorogenic acid (178.5 mg/100 g). After the storage period, fruit with cinnamon retained a comparable concentration of chlorogenic acid to convection dried fruit, which was previously soaked in water. Various kinds of fruit dried by convection did not differ statistically at a given measuring point, while the determined amount of chlorogenic acid in fruit dried by the combined method was placed between the amount in freeze-dried and convection dried fruit. After storage, chokeberry dried by the combined method contained the smallest amount of chlorogenic acid in fruit with the addition of cloves (188.3 mg/100 g), while in other fruit it amounted to 212.9–225.3 mg/100 g.

The expected influence of cinnamon or cloves on the content of polyphenols, particularly anthocyanins in chokeberry fruit was not observed. Nawirska-Olszańska, Pasławska, Stępień et al. [34] achieved a much higher level of preservation of anthocyanins, phenolic acids, flavonols, and procyanidins in chokeberry fruit impregnated with apple and pear juice, dried by the microwave-vacuum method. A relevant factor that influenced the obtained effect was the impregnation under pressure (4, 6, or 8 kPa).

The osmotic dehydration of a pumpkin with the use of chokeberry, flowering quince, or raspberry juice concentrates, followed by its vacuum-microwave drying, allowed for obtaining a dried pumpkin with much higher polyphenol content. The content of polyphenols in dried fruit increased with the time of osmotic pretreatment. The amount of polyphenol in a dried pumpkin was about 5–11 for chokeberry, 9–15.5 for flowering quince, and 2–3 times higher than the control sample [35].

### 3.6. The Influence of Plant Extracts on the Content Flavanols in Stored Dried Chokeberry Fruit

Storage of freeze-dried fruit has led to a reduction of epicatechin content in all types of fruit (Table 3). The level of losses in fruit with the addition of cinnamon (34.5%) and cloves (30.4%) was marginally different from the losses of epicatechin in fruit without additives (31.4%). The content of epicatechin in fruit dried by the combined method decreased as well. It was found that the loss of epicatechin in the combined method dried fruit, which was previously washed or soaked in water, cinnamon, and cloves amounted to 19.2, 11.6, 27.3, and 21.4%. There were no statistically significant differences between fruit dried by convection after three (33.67–37.81 mg/100 g), and nine months of storage (27.27–32.20 mg/100 g).

Changes in catechin content in freeze-dried fruit were statistically insignificant. Combined drying influenced the progressive loss of catechin concentration in fruit with the addition of cinnamon and cloves. Throughout the period of storage of fruit with spices, changes in the catechin content from 20.86 to 17.10 mg/100 g, and in the case of cinnamon, from 22.15 to 18.26 mg/100 g were recorded. Storage of convection dried fruit did not lead to any modifications in the level of catechin in fruit with the addition of cloves and cinnamon, which contained the amount of catechin similar to that from the beginning of the study.

### 3.7. The Influence of Plant Extracts on the Content Flavonols in Stored Dried Chokeberry Fruit

The conducted research resulted in establishing, that the storage of dried chokeberry fruit had a diverse influence on the content of flavonols (Table 4). The changes were very insignificant. During storage tests of dried fruit, no clear trend in quercetin glycosides concentration changes was observed. The maximum amount of loss after three and nine months of storage was recorded by quercetin-3-O-galactoside (5.72 mg/100 g) in fruit dried by the combination method with the addition of cloves, and quercetin-3-O-galactoside (6.64 mg/100 g) in freeze-dried fruit with cinnamon. The maximum increase of the concentration involved quercetin-3-O-galactoside (5.36 mg/100 g) in freeze-dried fruit washed after three months and quercetin-3-O-galactoside (4.06 mg/100 g) in washed and convection dried fruit after nine months of storage. There is currently no data on changes in the content of polyphenols in berries during the storage period. The available study, described below, relates to the storage of dried strawberries, raspberries, and bilberries. The process of storage resulted in losses of polyphenols and anthocyanins. The most significant damages were observed in bilberries, for which a sharp decrease in polyphenols was observed up to the sixth month (freeze-dried and air-dried fruit), as well as in anthocyanins, up to the second month of storage (freeze-dried fruit). During the following storage stages of bilberries and other fruit, the changes progressed gradually [36].

### 3.8. The Influence of Drying Method on the Content Polyphenols in Dried Chokeberry Fruit

The drying methods were compared in terms of the content of polyphenols, without taking into account the method of preparing the raw material for drying. It was found that the drying method had a significant effect on the polyphenol content in chokeberry fruit. The highest amount of polyphenols was found in freeze-dried fruits 3385 mg/100 g, their amount was about 3 times higher than the number of polyphenols in fruits dried using the combined method 1131 mg/100 g and convection method 1102 mg/100 g (*p* < 0.05).

It was confirmed that the above dependence was mainly determined by anthocyanins, which were present in fruit dried by freeze-drying, convection, and the combined method at the level of 2717, 611.1, and 548.5 mg/100 g, respectively. The differences in the content of phenolic acids in dried fruit were lower but statistically significant. The content of phenolic acids was as follows: freeze-drying 486.3 mg/100 g > combined method 421.7 mg/100 g > convection method 364.0 mg/100 g. The freeze-drying process also influenced significantly greater preservation of flavanols (77.18 mg/100 g). In fruit dried using the combined method, 65.10 mg/100 g were determined, statistically more flavonols than in convection-dried fruit (50.45 mg/100 g). The fruit dried by freeze-drying and the combined method had a similar content of flavonols (104.1 and 95.48 mg/100 g), while the fruit droughts obtained by the convection method contained significantly less amount of those components (76.15 mg/100 g).

## 4. Conclusions

The most desirable drying method which preserves the largest amount of polyphenols is freeze-drying. Combined or convection drying at 80 °C resulted in significantly higher losses of polyphenols, particularly anthocyanins. Cinnamon or cloves added to the fruit prior to drying did not have a positive influence on its composition. The additives did not affect the content of phenolic acids, flavanols, and flavonols. However, during the process of combined and convective drying, yet not freeze-drying, they led to a decrease in the level of anthocyanin. Cinnamon and cloves accelerated the loss of anthocyanin during storage. Storage of dried fruit with the addition of cloves resulted in higher losses of neochlorogenic acid. The results obtained in the study show that the most favorable composition of biologically active compounds such as polyphenols is achieved after the freeze-drying process. It was also pointed out that dry snacks or chokeberry chips should not be dried after soaking in spice solutions such as cinnamon and clove, as they accelerate the degradation of anthocyanins. The research demonstrates that the discovery of an appropriate additive in order to preserve polyphenols is not an easy task. Moreover, there is also a need to consider the method of applying additives to fruit prior to drying. However, it is worth noting that the content of health-promoting compounds indicates that dried black chokeberry fruits may be used for the production of functional foods in the form of chips or as a food additive aimed at improving the biological value of food.

## Figures and Tables

**Table 1:** Changes in anthocyanins content during storage of tested black chokeberry fruits.

		Dried Fruit		
		Storage (month)		
Drying Method	Additive	0	3	9
cyanidin-3-*O*-galactoside (mg/100 g)				
Freeze drying	No preparation	1487.26 ± 70.72 ^A,a^	1533.71 ± 60.78 ^A,a^	1053.01 ± 1.00 ^B,a^
	Soaked in water	1403.05 ± 33.10 ^A,a^	1400.54 ± 28.10 ^A,ab^	907.08 ± 18.16 ^B,ab^
	Soaked in cinnamon suspension	1433.04 ± 94.06 ^A,a^	1301.23 ± 104.05 ^A,ab^	865.20 ± 5.95 ^B,b^
	Soaked in clove suspension	1587.23 ± 120.20 ^A,a^	1168.65 ± 28.94 ^B,b^	901.86 ± 86.27 ^B,ab^
Combined drying	No preparation	415.76 ± 34.03 ^A,a^	349.72 ± 8.43 ^A,a^	190.88 ± 4.97 ^B,a^
	Soaked in water	300.26 ± 9.14 ^A,b^	282.26 ± 1.46 ^A,b^	149.93 ± 2.63 ^B,b^
	Soaked in cinnamon suspension	317.77 ± 15.87 ^A,b^	256.49 ± 1.58 ^B,c^	130.52 ± 8.25 ^C,b^
	Soaked in clove suspension	254.03 ± 0.40 ^A,b^	191.84 ± 2.56 ^B,d^	99.84 ± 9.74 ^C,c^
Convective drying	No preparation	438.19 ± 28.38 ^A,a^	420.06 ± 0.10 ^A,a^	208.21 ± 6.77 ^B,a^
	Soaked in water	388.40 ± 8.45 ^A,ab^	345.66 ± 10.08 ^B,b^	174.40 ± 1.81 ^C,a^
	Soaked in cinnamon suspension	292.66 ± 21.20 ^A,c^	275.60 ± 0.40 ^A,c^	124.37 ± 20.18 ^B,b^
	Soaked in clove suspension	328.66 ± 32.35 ^A,c^	257.83 ± 2.35 ^A,c^	123.59 ± 18.69 ^B,b^
cyanidin-3-*O*-glucoside (mg/100 g)				
Freeze drying	No preparation	52.53 ± 3.19 ^A,a^	55.62 ± 0.17 ^A,a^	29.76 ± 0.13 ^B,a^
	Soaked in water	54.40 ± 3.93 ^A,a^	52.18 ± 3.69 ^A,ab^	24.71 ± 2.83 ^B,a^
	Soaked in cinnamon suspension	51.14 ± 4.99 ^A,a^	43.80 ± 3.39 ^A,bc^	22.97 ± 0.13 ^B,a^
	Soaked in clove suspension	60.68 ± 1.99 ^A,a^	38.01 ± 1.02 ^B,c^	25.17 ± 2.65 ^C,a^
Combined drying	No preparation	11.39 ± 0.55 ^A,a^	10.36 ± 0.58 ^A,a^	5.07 ± 0.22 ^B,a^
	Soaked in water	9.31 ± 0.06 ^A,b^	8.12 ± 0.02 ^B,b^	4.25 ± 0.03 ^C,ab^
	Soaked in cinnamon suspension	9.16 ± 0.08 ^A,b^	7.16 ± 0.26 ^B,b^	3.56 ± 0.24 ^C,bc^
	Soaked in clove suspension	7.34 ± 0.23 ^A,c^	5.75 ± 0.16 ^B,c^	2.75 ± 0.41 ^C,c^
Convective drying	No preparation	12.17 ± 0.72 ^A,a^	12.02 ± 0.76 ^A,a^	5.46 ± 0.13 ^B,a^
	Soaked in water	10.97 ± 0.15 ^A,ab^	9.50 ± 0.28 ^B,b^	4.70 ± 0.13 ^C,a^
	Soaked in cinnamon suspension	8.33 ± 0.92 ^A,b^	7.65 ± 0.22 ^A,b^	3.16 ± 0.63 ^B,b^
	Soaked in clove suspension	9.34 ± 0.64 ^A,b^	7.58 ± 0.58 ^A,b^	3.41 ± 0.30 ^B,b^
cyanidin-3-*O*-arabinoside [mg/100 g]				
Freeze drying	No preparation	1053.45 ± 45.27 ^A,a^	1079.38 ± 60.62 ^A,a^	651.55 ± 4.51 ^B,a^
	Soaked in water	1047.40 ± 65.83 ^A,a^	994.35 ± 57.15 ^A,a^	541.17 ± 25.46 ^B,b^
	Soaked in cinnamon suspension	996.07 ± 57.46 ^A,a^	888.36 ± 45.11 ^A,ab^	510.51 ± 12.04 ^B,b^
	Soaked in clove suspension	1112.08 ± 27.11 ^A,a^	742.00 ± 5.90 ^B,b^	540.58 ± 32.03 ^C,b^
Combined drying	No preparation	242.97 ± 13.33 ^A,a^	215.10 ± 11.03 ^A,a^	109.76 ± 3.72 ^B,a^
	Soaked in water	185.86 ± 1.52 ^A,b^	169.53 ± 0.85 ^B,b^	90.26 ± 2.18 ^C,b^
	Soaked in cinnamon suspension	192.79 ± 5.17 ^A,b^	152.76 ± 3.22 ^B,b^	79.78 ± 3.31 ^C,b^
	Soaked in clove suspension	164.12 ± 3.80 ^A,b^	117.40 ± 1.32 ^B,c^	62.23 ± 5.85 ^C,c^
Convective drying	No preparation	251.68 ± 11.23 ^A,a^	250.78 ± 7.46 ^A,a^	122.23 ± 2.55 ^B,a^
	Soaked in water	239.19 ± 5.06 ^A,ab^	201.49 ± 7.36 ^B,b^	106.04 ± 1.34 ^C,a^
	Soaked in cinnamon suspension	180.60 ± 18.89 ^A,b^	163.80 ± 2.47 ^A,c^	73.77 ± 11.19 ^B,b^
	Soaked in clove suspension	191.87 ± 18.56 ^A,b^	150.98 ± 3.76 ^A,c^	77.64 ± 6.85 ^B,b^
cyanidin-3-*O*-xyloside [mg/100 g]				
Freeze drying	No preparation	134.29 ± 2.29 ^A,a^	136.79 ± 12.68 ^A,a^	73.18 ± 0.83 ^B,a^
	Soaked in water	139.62 ± 12.36 ^A,a^	116.23 ± 7.30 ^A,a^	69.59 ± 5.40 ^B,a^
	Soaked in cinnamon suspension	120.80 ± 6.19 ^A,a^	108.10 ± 3.45 ^A,a^	52.85 ± 0.70 ^B,b^
	Soaked in clove suspension	135.00 ± 0.07 ^A,a^	103.23 ± 10.16 ^B,a^	64.81 ± 3.41 ^C,ab^
Combined drying	No preparation	24.50 ± 2.15 ^A,a^	21.37 ± 0.85 ^A,a^	10.52 ± 0.40 ^B,a^
	Soaked in water	19.97 ± 0.14 ^A,ab^	17.07 ± 0.54 ^B,b^	8.92 ± 0.31 ^C,ab^
	Soaked in cinnamon suspension	21.39 ± 0.02 ^A,ab^	15.94 ± 0.24 ^B,b^	8.02 ± 0.67 ^C,b^
	Soaked in clove suspension	17.44 ± 1.22 ^A,b^	12.13 ± 0.36 ^B,c^	6.27 ± 0.16 ^C,c^
Convective drying	No preparation	29.70 ± 3.09 ^A,a^	25.21 ± 0.98 ^A,a^	12.57 ± 0.22 ^B,a^
	Soaked in water	24.19 ± 0.10 ^A,a^	20.79 ± 0.85 ^B,b^	10.42 ± 0.27 ^C,b^
	Soaked in cinnamon suspension	18.06 ± 2.39 ^A,b^	17.02 ± 1.61 ^A,bc^	7.13 ± 0.99 ^B,c^
	Soaked in clove suspension	20.42 ± 1.06 ^A,b^	16.33 ± 0.26 ^B,c^	7.42 ± 0.54 ^C,c^

a, b, c—means in a column followed by the same small letter are not significantly different (*p* > 0.05); A, B, C—means in a row followed by the same capital letter are not significantly different (*p* > 0.05); values are means of three determinations ± SD.

**Table 2:** Changes in phenolic acids content during storage of tested black chokeberry fruits.

		Dried Fruit		
		Storage (month)		
Drying Method	Additive	0	3	9
neochlorogenic acid [mg/100 g]				
Freeze drying	No preparation	208.89 ± 3.75 ^A,a^	212.86 ± 2.03 ^A,a^	175.62 ± 1.34 ^B,a^
	Soaked in water	207.47 ± 1.13 ^A,a^	191.98 ± 1.29 ^B,b^	153.94 ± 4.32 ^C,c^
	Soaked in cinnamon suspension	199.93 ± 3.60 ^A,a^	192.28 ± 2.21 ^A,b^	168.46 ± 2.64 ^B,ab^
	Soaked in clove suspension	199.57 ± 1.00 ^A,a^	172.40 ± 0.54 ^B,c^	163.70 ± 2.18 ^C,bc^
Combined drying	No preparation	179.18 ± 10.23 ^A,a^	176.77 ± 8.55 ^A,a^	149.78 ± 2.51 ^A,ab^
	Soaked in water	161.89 ± 1.26 ^A,a^	169.65 ± 2.18 ^A,a^	141.34 ± 3.30 ^B,ab^
	Soaked in cinnamon suspension	173.10 ± 9.13 ^A,a^	160.36 ± 0.58 ^AB,a^	134.80 ± 5.99 ^B,bc^
	Soaked in clove suspension	159.97 ± 1.28 ^A,a^	138.91 ± 1.10 ^B,b^	123.11 ± 0.17 ^C,c^
Convective drying	No preparation	158.44 ± 13.13 ^A,a^	176.47 ± 4.08 ^A,a^	146.22 ± 4.56 ^A,a^
	Soaked in water	161.55 ± 1.64 ^A,a^	173.57 ± 4.24 ^A,a^	130.15 ± 9.27 ^B,ab^
	Soaked in cinnamon suspension	142.38 ± 11.51 ^A,a^	157.73 ± 2.95 ^A,b^	134.18 ± 2.27 ^A,ab^
	Soaked in clove suspension	144.37 ± 0.02 ^A,a^	147.07 ± 1.58 ^A,b^	121.00 ± 5.59 ^B,b^
chlorogenic acid (mg/100 g)				
Freeze drying	No preparation	293.26 ± 9.78 ^A,a^	306.64 ± 0.63 ^A,a^	249.76 ± 3.05 ^B,a^
	Soaked in water	284.21 ± 5.67 ^A,a^	268.70 ± 6.16 ^AB,b^	232.86 ± 14.21 ^B,a^
	Soaked in cinnamon suspension	280.77 ± 5.45 ^A,a^	262.13 ± 4.45 ^A,b^	238.30 ± 3.75 ^B,a^
	Soaked in clove suspension	271.14 ± 3.12 ^A,a^	234.87 ± 5.86 ^B,c^	233.92 ± 0.92 ^B,a^
Combined drying	No preparation	259.03 ± 15.13 ^A,ab^	253.20 ± 8.04 ^A,a^	219.26 ± 2.93 ^A,a^
	Soaked in water	243.78 ± 3.83 ^A,ab^	244.31 ± 8.28 ^A,a^	213.84 ± 2.74 ^B,a^
	Soaked in cinnamon suspension	273.18 ± 8.22 ^A,a^	239.57 ± 0.70 ^B,a^	212.86 ± 11.07 ^B,ab^
	Soaked in clove suspension	236.68 ± 1.08 ^A,b^	206.09 ± 1.40 ^B,b^	188.28 ± 3.79 ^C,b^
Convective drying	No preparation	225.58 ± 15.35 ^A,a^	251.46 ± 0.03 ^A,a^	218.72 ± 2.19 ^A,a^
	Soaked in water	222.56 ± 0.32 ^A,a^	244.44 ± 6.69 ^A,a^	191.29 ± 12.37 ^B,ab^
	Soaked in cinnamon suspension	200.81 ± 18.57 ^A,a^	233.26 ± 9.97 ^A,ab^	195.49 ± 5.13 ^A,ab^
	Soaked in clove suspension	200.15 ± 1.54 ^A,a^	213.85 ± 5.61 ^A,b^	178.45 ± 16.19 ^A,b^

a, b, c—means in a column followed by the same small letter are not significantly different (*p* > 0.05); A, B, C—means in a row followed by the same capital letter are not significantly different (*p* > 0.05); values are means of three determinations ± SD.

**Table 3:** Changes in flavanols content during storage of tested black chokeberry fruits.

		Dried Fruit		
		Storage (month)		
Drying Method	Additive	0	3	9
(+)-catechin (mg/100 g)				
Freeze drying	No preparation	11.95 ± 0.06 ^A,a^	15.44 ± 4.47 ^A,a^	14.54 ± 0.14 ^A,ab^
	Soaked in water	14.89 ± 4.01 ^A,a^	11.55 ± 0.95 ^A,a^	13.35 ± 0.89 ^A,b^
	Soaked in cinnamon suspension	15.14 ± 3.61 ^A,a^	14.22 ± 3.61 ^A,a^	14.64 ± 0.73 ^A,ab^
	Soaked in clove suspension	19.82 ± 0.09 ^A,a^	16.16 ± 2.59 ^A,a^	16.93 ± 0.62 ^A,a^
Combined drying	No preparation	23.01 ± 3.32 ^A,a^	21.18 ± 1.35 ^A,a^	20.22 ± 0.67 ^A,a^
	Soaked in water	19.57 ± 1.03 ^A,a^	20.83 ± 0.10 ^A,a^	18.76 ± 0.67 ^A,ab^
	Soaked in cinnamon suspension	20.86 ± 0.16 ^A,a^	19.49 ± 0.44 ^B,a^	17.10 ± 0.22 ^C,b^
	Soaked in clove suspension	22.15 ± 1.04 ^A,a^	20.39 ± 0.20 ^AB,a^	18.26 ± 0.69 ^B,ab^
Convective drying	No preparation	17.40 ± 3.59 ^A,a^	17.77 ± 0.31 ^A,a^	17.40 ± 0.24 ^A,a^
	Soaked in water	15.14 ± 0.30 ^B,a^	17.21 ± 0.40 ^A,a^	15.53 ± 0.57 ^B,b^
	Soaked in cinnamon suspension	13.29 ± 0.98 ^A,a^	15.40 ± 0.34 ^A,b^	13.80 ± 0.01 ^A,c^
	Soaked in clove suspension	16.05 ± 0.86 ^A,a^	17.94 ± 0.27 ^A,a^	15.23 ± 0.89 ^A,bc^
(-)-epicatechin (mg/100 g)				
Freeze drying	No preparation	62.94 ± 2.46 ^A,a^	57.85 ± 0.12 ^A,a^	43.18 ± 0.16 ^B,a^
	Soaked in water	60.47 ± 0.97 ^A,a^	57.20 ± 0.19 ^A,a^	41.03 ± 1.28 ^B,a^
	Soaked in cinnamon suspension	60.26 ± 0.54 ^A,a^	53.83 ± 0.37 ^B,b^	39.23 ± 0.28 ^C,a^
	Soaked in clove suspension	63.26 ± 3.32 ^A,a^	49.99 ± 1.36 ^B,c^	44.01 ± 2.29 ^B,a^
Combined drying	No preparation	46.29 ± 5.94 ^A,a^	40.83 ± 0.90 ^A,a^	37.40 ± 0.05 ^A,a^
	Soaked in water	40.80 ± 0.35 ^A,a^	39.62 ± 1.19 ^AB,a^	36.08 ± 0.97 ^B,ab^
	Soaked in cinnamon suspension	44.30 ± 2.22 ^A,a^	40.72 ± 0.68 ^A,a^	32.21 ± 1.54 ^B,b^
	Soaked in clove suspension	43.39 ± 0.13 ^A,a^	38.11 ± 0.86 ^B,a^	34.12 ± 1.53 ^B,ab^
Convective drying	No preparation	37.56 ± 3.62 ^A,a^	37.35 ± 2.12 ^A,a^	32.22 ± 0.93 ^A,a^
	Soaked in water	35.08 ± 0.39 ^B,a^	37.82 ± 1.04 ^A,a^	30.82 ± 0.86 ^C,a^
	Soaked in cinnamon suspension	30.77 ± 1.43 ^AB,a^	33.67 ± 1.91 ^A,a^	27.27 ± 0.50 ^B,a^
	Soaked in clove suspension	36.51 ± 1.51 ^A,a^	37.72 ± 0.70 ^A,a^	30.51 ± 4.24 ^A,a^

a, b, c—means in a column followed by the same small letter are not significantly different (*p* > 0.05); A, B, C—means in a row followed by the same capital letter are not significantly different (*p* > 0.05); values are means of three determinations ± SD.

**Table 4:** Changes in flavonols content during storage of tested black chokeberry fruits.

		Dried Fruit		
		Storage (month)		
Drying Method	Additive	0	3	9
quercetin-3-*O*-vicianoside (mg/100 g)				
Freeze drying	No preparation	20.10 ± 0.19 ^A,a^	21.78 ± 1.96 ^A,a^	17.83 ± 0.14 ^A,ab^
	Soaked in water	21.10 ± 0.48 ^A,a^	17.53 ± 0.62 ^B,a^	17.99 ± 0.14 ^B,ab^
	Soaked in cinnamon suspension	19.66 ± 0.48 ^A,a^	17.37 ± 0.32 ^B,a^	15.93 ± 0.19 ^B,b^
	Soaked in clove suspension	19.86 ± 0.62 ^A,a^	19.76 ± 1.96 ^A,a^	19.26 ± 1.61 ^A,a^
Combined drying	No preparation	20.05 ± 2.00 ^A,a^	18.11 ± 0.10 ^A,a^	17.99 ± 0.50 ^A,a^
	Soaked in water	19.07 ± 0.69 ^A,a^	17.97 ± 0.38 ^A,a^	17.81 ± 0.88 ^A,a^
	Soaked in cinnamon suspension	18.45 ± 0.16 ^A,a^	16.51 ± 0.30 ^B,b^	16.80 ± 0.44 ^B,a^
	Soaked in clove suspension	19.28 ± 0.88 ^A,a^	16.13 ± 0.32 ^B,b^	17.31 ± 0.90 ^AB,a^
Convective drying	No preparation	15.44 ± 1.59 ^A,a^	18.70 ± 1.35 ^A,a^	18.82 ± 1.24 ^A,a^
	Soaked in water	15.62 ± 1.06 ^AB,a^	16.94 ± 0.43 ^A,a^	14.74 ± 0.65 ^B,b^
	Soaked in cinnamon suspension	13.57 ± 1.99 ^A,a^	16.88 ± 0.11 ^A,a^	14.10 ± 0.85 ^A,b^
	Soaked in clove suspension	15.12 ± 0.37 ^A,a^	17.21 ± 1.55 ^A,a^	14.74 ± 0.87 ^A,b^
quercetin-3-*O*-glucoside (mg/100 g)				
Freeze drying	No preparation	23.35 ± 0.04 ^A,bc^	25.87 ± 0.67 ^A,a^	22.16 ± 1.46 ^A,ab^
	Soaked in water	25.14 ± 0.05 ^A,b^	22.02 ± 1.11 ^B,ab^	21.36 ± 0.50 ^B,ab^
	Soaked in cinnamon suspension	23.08 ± 0.91 ^A,c^	21.10 ± 0.97 ^AB,c^	19.14 ± 0.11 ^B,b^
	Soaked in clove suspension	27.20 ± 0.35 ^A,a^	23.53 ± 1.22 ^A,a^	23.82 ± 1.54 ^A,a^
Combined drying	No preparation	24.11 ± 1.89 ^A,a^	22.85 ± 0.84 ^A,a^	22.54 ± 0.10 ^A,a^
	Soaked in water	21.94 ± 0.62 ^A,a^	21.82 ± 0.12 ^A,a^	21.72 ± 0.67 ^A,a^
	Soaked in cinnamon suspension	21.37 ± 0.99 ^A,a^	19.24 ± 0.14 ^A,b^	18.92 ± 0.38 ^A,b^
	Soaked in clove suspension	22.30 ± 1.62 ^A,a^	19.54 ± 0.18 ^A,b^	20.98 ± 0.48 ^A,a^
Convective drying	No preparation	19.68 ± 1.17 ^B,a^	23.14 ± 0.58 ^A,a^	21.79 ± 0.48 ^AB,a^
	Soaked in water	19.74 ± 0.88 ^AB,a^	21.32 ± 0.52 ^A,ab^	18.91 ± 0.78 ^B,b^
	Soaked in cinnamon suspension	16.22 ± 2.15 ^A,a^	18.95 ± 0.21 ^A,c^	16.78 ± 0.84 ^A,b^
	Soaked in clove suspension	18.75 ± 0.70 ^A,a^	20.92 ± 0.73 ^A,bc^	18.03 ± 1.65 ^A,b^
quercetin-3-*O*-galactoside (mg/100 g)				
Freeze drying	No preparation	32.91 ± 0.93 ^A,a^	38.27 ± 3.15 ^A,a^	31.12 ± 0.70 ^A,a^
	Soaked in water	35.22 ± 0.07 ^A,a^	30.01 ± 0.85 ^B,a^	30.43 ± 1.04 ^B,a^
	Soaked in cinnamon suspension	33.41 ± 2.10 ^A,a^	29.49 ± 1.92 ^A,a^	26.77 ± 0.45 ^A,a^
	Soaked in clove suspension	36.93 ± 1.49 ^A,a^	34.67 ± 4.47 ^A,a^	34.13 ± 3.84 ^A,a^
Combined drying	No preparation	34.49 ± 4.38 ^A,a^	30.04 ± 0.87 ^A,a^	30.50 ± 0.68 ^A,a^
	Soaked in water	29.33 ± 0.83 ^A,a^	28.99 ± 0.21 ^A,a^	29.15 ± 1.40 ^A,ab^
	Soaked in cinnamon suspension	29.28 ± 0.67 ^A,a^	25.36 ± 0.10 ^B,b^	25.75 ± 0.72 ^B,b^
	Soaked in clove suspension	30.40 ± 1.98 ^A,a^	24.68 ± 0.45 ^A,b^	26.16 ± 1.26 ^A,b^
Convective drying	No preparation	26.16 ± 2.68 ^A,a^	30.46 ± 1.73 ^A,a^	30.22 ± 0.96 ^A,a^
	Soaked in water	26.08 ± 2.28 ^A,a^	27.75 ± 0.23 ^A,ab^	24.79 ± 1.55 ^A,b^
	Soaked in cinnamon suspension	20.58 ± 2.87 ^A,a^	25.00 ± 0.75 ^A,b^	20.77 ± 1.21 ^A,b^
	Soaked in clove suspension	23.39 ± 1.61 ^A,a^	27.42 ± 1.41 ^A,ab^	22.65 ± 2.44 ^A,b^
quercetin-3-*O*-rutinoside (mg/100 g)				
Freeze drying	No preparation	23.26 ± 0.09 ^A,a^	25.84 ± 1.94 ^A,a^	22.44 ± 1.28 ^A,ab^
	Soaked in water	24.74 ± 0.23 ^A,a^	21.27 ± 0.82 ^B,a^	20.52 ± 1.04 ^B,ab^
	Soaked in cinnamon suspension	23.31 ± 2.14 ^A,a^	20.69 ± 1.43 ^A,a^	18.59 ± 0.09 ^A,b^
	Soaked in clove suspension	26.98 ± 0.34 ^A,a^	23.43 ± 1.36 ^A,a^	23.86 ± 1.63 ^A,a^
Combined drying	No preparation	23.74 ± 3.13 ^A,a^	21.74 ± 0.97 ^A,a^	23.05 ± 0.54 ^A,a^
	Soaked in water	22.13 ± 0.77 ^A,a^	21.40 ± 1.05 ^A,a^	22.38 ± 1.07 ^A,a^
	Soaked in cinnamon suspension	22.78 ± 0.75 ^A,a^	20.47 ± 0.42 ^A,a^	20.18 ± 0.96 ^A,a^
	Soaked in clove suspension	23.20 ± 1.40 ^A,a^	20.17 ± 0.26 ^A,a^	22.25 ± 0.97 ^A,a^
Convective drying	No preparation	19.28 ± 1.88 ^A,a^	21.92 ± 1.55 ^A,a^	21.72 ± 0.46 ^A,a^
	Soaked in water	18.92 ± 1.30 ^A,a^	21.56 ± 0.71 ^A,a^	18.34 ± 1.22 ^A,a^
	Soaked in cinnamon suspension	16.48 ± 2.13 ^A,a^	20.20 ± 0.70 ^A,a^	17.73 ± 1.45 ^A,a^
	Soaked in clove suspension	19.57 ± 0.31 ^A,a^	21.43 ± 2.23 ^A,a^	18.41 ± 1.35 ^A,a^

a, b, c—means in a column followed by the same small letter are not significantly different (*p* > 0.05); A, B—means in a row followed by the same capital letter are not significantly different (*p* > 0.05); values are means of three determinations ± SD.

**Table 5:** Equation of the curve for degradation, constant reaction rate, and half-life of anthocyanins in dried chokeberry fruits.

Drying Method	Fruits	Equation for the Curve of Logarithm of Substrate Concentration Versus Time *t* (d) for First Order Reactions	R^2^	*k* (d^−1^)	t_1/2_ (d)
cyanidin-3-*O*-galactoside [mg/100 g]					
Freeze drying	No preparation	y = −0.0006x + 3.1995	0.8432	0.6 × 10^−3^	1155.0
	Soaked in water	y = −0.0008x + 3.1738	0.9913	0.8 × 10^−3^	866.3
	Soaked in cinnamon suspension	y = −0.0008x + 3.1696	0..9770	0.8 × 10^−3^	866.3
	Soaked in clove suspension	y = −0.0009x + 3.1787	0..9444	0.9 × 10^−3^	770.0
Combined drying	No preparation	y = −0.0013x + 2.6349	0.9856	1.3 × 10^−3^	533.1
	Soaked in water	y = −0.0012x + 2.5091	0.9899	1.2 × 10^−3^	577.5
	Soaked in cinnamon suspension	y = −0.0015x + 2.5174	0.9899	1.5 × 10^−3^	462.0
	Soaked in clove suspension	y = −0.0015x + 2.4106	0.9987	1.5 × 10^−3^	462.0
Convective drying	No preparation	y = −0.0013x + 2.6800	0.9221	1.3 × 10^−3^	533.1
	Soaked in water	y = −0.0013x + 2.6173	0.9612	1.3 × 10^−3^	533.1
	Soaked in cinnamon suspension	y = −0.0015x + 2.5083	0.9286	1.5 × 10^−3^	462.0
	Soaked in clove suspension	y = −0.0016x + 2.5322	0.9914	1.6 × 10^−3^	433.1
cyanidin-3-*O*-glucoside (mg/100 g)					
Freeze drying	No preparation	y = −0.0010x + 1.7663	0.8366	1.0 × 10^−3^	693.0
	Soaked in water	y = −0.0013x + 1.7768	0.9201	1.3 × 10^−3^	533.1
	Soaked in cinnamon suspension	y = −0.0013x + 1.7296	0.9777	1.3 × 10^−3^	533.1
	Soaked in clove suspension	y = −0.0014x +1.7506	0.9494	1.4 × 10^−3^	495.0
Combined drying	No preparation	y = −0.0014x + 1.0891	0.9494	1.4 × 10^−3^	495.0
	Soaked in water	y = −0.0013x + 0.9920	0.9714	1.3 × 10^−3^	533.1
	Soaked in cinnamon suspension	y = −0.0015x + 0.9748	0.9937	1.5 × 10^−3^	462.0
	Soaked in clove suspension	y = −0.0016x + 0.8815	0.9915	1.6 × 10^−3^	433.1
Convective drying	No preparation	y = −0.0014x + 1.1325	0.9013	1.4 × 10^−3^	495.0
	Soaked in water	y = −0.0014x + 1.0660	0.9698	1.4 × 10^−3^	495.0
	Soaked in cinnamon suspension	y = −0.0016x + 0.9647	0.9370	1.6 × 10^−3^	433.1
	Soaked in clove suspension	y = −0.0017x + 0.9942	0.9817	1.7 × 10^−3^	407.6
cyanidin-3-*O*-arabinoside (mg/100 g)					
Freeze drying	No preparation	y = −0..0008x + 3.0569	0.8650	0.8 × 10^−3^	866.3
	Soaked in water	y = −0.0011x + 3.0514	0.9326	1.1 × 10^−3^	630.0
	Soaked in cinnamon suspension	y = −0.0011x + 3.0185	0.9705	1.1 × 10^−3^	630.0
	Soaked in clove suspension	y = −0.0011x + 3.0156	0.9337	1.1 × 10^−3^	630.0
Combined drying	No preparation	y = −0.0013x + 2.4122	0.9641	1.3 × 10^−3^	533.1
	Soaked in water	y = −0.0012x + 2.2969	0.9540	1.2 × 10^−3^	577.5
	Soaked in cinnamon suspension	y = −0.0014x + 2.2965	0.9942	1.4 × 10^−3^	495.0
	Soaked in clove suspension	y = −0.0016x + 2.2130	0.9998	1.6 × 10^−3^	433.1
Convective drying	No preparation	y = −0.0012x + 2.4450	0.8955	1.2 × 10^−3^	577.5
	Soaked in water	y = −0.0013x + 2.3973	0.9826	1.3 × 10^−3^	533.1
	Soaked in cinnamon suspension	y = −0.0015x + 2.2941	0.9463	1.5 × 10^−3^	462.0
	Soaked in clove suspension	y = −0.0015x + 2.2945	0.9944	1.5 × 10^−3^	462.0
cyanidin-3-*O*-xyloside (mg/100 g)					
Freeze drying	No preparation	y = −0.0011x + 2.1691	0.8763	1.1 × 10^−3^	630.0
	Soaked in water	y = −0.0011x + 2.1540	0.9941	1.1 × 10^−3^	630.0
	Soaked in cinnamon suspension	y = −0.0014x + 2.1127	0.9568	1.4 × 10^−3^	495.0
	Soaked in clove suspension	y = −0.0012x + 2.1259	0.9987	1.2 × 10^−3^	577.5
Combined drying	No preparation	y = −0.0014x + 1.4161	0.9671	1.4 × 10^−3^	495.0
	Soaked in water	y = −0.0015x + 1.3845	0.9415	1.5 × 10^−3^	462.0
	Soaked in cinnamon suspension	y = −0.0016x + 1.3364	0.9986	1.6 × 10^−3^	433.1
	Soaked in clove suspension	y = −0.0016x + 1.2375	0.9994	1.6 × 10^−3^	433.1
Convective drying	No preparation	y = −0.0014x + 1.4956	0.9768	1.4 × 10^−3^	495.0
	Soaked in water	y = −0.0014x + 1.4077	0.9734	1.4 × 10^−3^	495.0
	Soaked in cinnamon suspension	y = −0.0017x + 1.3313	0.9852	1.7 × 10^−3^	407.6
	Soaked in clove suspension	y = −0.0016x + 1.3033	0.9256	1.6 × 10^−3^	433.1

## Data Availability

Data is contained within the article.
